# The Transformation of Reference Microbiology Methods and Surveillance for *Salmonella* With the Use of Whole Genome Sequencing in England and Wales

**DOI:** 10.3389/fpubh.2019.00317

**Published:** 2019-11-21

**Authors:** Marie Anne Chattaway, Timothy J. Dallman, Lesley Larkin, Satheesh Nair, Jacquelyn McCormick, Amy Mikhail, Hassan Hartman, Gauri Godbole, David Powell, Martin Day, Robert Smith, Kathie Grant

**Affiliations:** ^1^Gastrointestinal Bacteria Reference Unit, Public Health England, London, United Kingdom; ^2^Tuberculosis, Acute Respiratory, Gastrointestinal, Emerging/Zoonotic Infections, and Travel Health and IHR Division (T.A.R.G.E.T.), Public Health England, London, United Kingdom; ^3^Public Health Wales, Cardiff, United Kingdom

**Keywords:** WGS, genomic typing, molecular epidemiology, *Salmonella*, SNP typing

## Abstract

The use of whole genome sequencing (WGS) as a method for supporting outbreak investigations, studying *Salmonella* microbial populations and improving understanding of pathogenicity has been well-described (1–3). However, performing WGS on a discrete dataset does not pose the same challenges as implementing WGS as a routine, reference microbiology service for public health surveillance. Challenges include translating WGS data into a useable format for laboratory reporting, clinical case management, *Salmonella* surveillance, and outbreak investigation as well as meeting the requirement to communicate that information in an understandable and universal language for clinical and public health action. Public Health England have been routinely sequencing all referred presumptive *Salmonella* isolates since 2014 which has transformed our approach to reference microbiology and surveillance. Here we describe an overview of the integrated methods for cross-disciplinary working, describe the challenges and provide a perspective on how WGS has impacted the laboratory and surveillance processes in England and Wales.

## Introduction

Public Health England's (PHE) Gastrointestinal Bacterial Reference Unit (GBRU) receives approximately 10,000 presumptive *Salmonella* isolates each year from diagnostic microbiology laboratories, private laboratories and food, water and environmental laboratories for confirmation of identity and typing. Of the average 8,500 individual case reports of salmonellosis in England and Wales annually, ~95% of clinical diagnostic isolates are sent to the reference laboratory for confirmation and further typing. The reporting of *Salmonella* isolated from human clinical diagnostic samples in public health laboratories is mandatory under national legislation (4, 5).

Prior to the introduction of WGS, presumptive *Salmonella* isolates were identified and characterized using a variety of methods including assaying biochemical properties (6), real-time PCR (7), phenotypic microarrays (Omnilog), and serology (8, 9). Further discrimination for select serovars was routinely carried out using phage-typing (PT) (10) and suspected outbreak isolates were reactively subjected to pulsed-field gel electrophoresis (PFGE) (11) or multi-locus variable number of tandem repeats analysis (MLVA) (12). The approach of using multiple laboratory techniques for the characterization of *Salmonella* was highly specialized, laborious, time consuming and open to interpretation error. When the option of using a Whole Genome Sequencing (WGS) approach to streamline laboratory processes, reduce processing time, improve the fine typing discriminatory power for surveillance and outbreak detection in real-time became available, PHE utilized the opportunity to assess its potential in a public health setting.

In 2014, GBRU began evaluating and validating WGS methods as a replacement for conventional confirmation and further characterization methods for *Salmonella* spp and began reporting results derived from WGS analysis routinely for surveillance purposes from April 2015 (13). The implementation of this methodology has required a change in how we approach our testing processes, the reporting of microbiological data, the integration with epidemiological data and application of cross-disciplinary working encompassing microbiological, bioinformatics and epidemiological expertise. Here, following 4 full years of implementation in England and Wales, we describe an overview of our experiences to date, provide a perspective on our approach to maximize the utility and benefits, present on overview of WGS data generated between April 2016 and March 2018 and describe some of the limitations and challenges in implementing WGS for routine *Salmonella* surveillance.

## PHEs WGS Implementation Approach

### Identification of *Salmonella* and the Bioinformatics Pipeline Process

**Figure 1:**
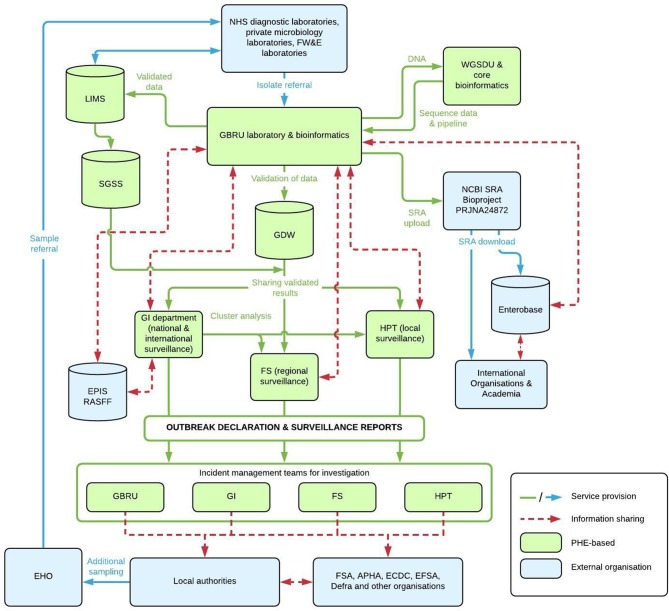
Flow Chart of Service Provision and information workflows between PHE and external organizations for *Salmonella* reference microbiology and surveillance. NHS, National Health Service; FW&E, Food, Water and Environmental; PHE, Public Health England; GBRU, Gastrointestinal Bacteria Reference Unit; WGSDU, Whole Genome Sequencing Delivery Unit; LIMS, Laboratory Information Management System; GDW, Gastro Data Warehouse; SGSS, Second Generation Surveillance System; NCBI, National Center for Biotechnology Information; SRA, Short Read Archive; GI, Gastrointestinal; FS, Field Services; HPT, Health Protection Team; EPIS, Epidemic Intelligence Information System; RASFF, Rapid Alert System for Food and Feed; EHO, Environmental Health Officers; FSA, Food Standards Agency; APHA, Animal and Plant Health Agency; ECDC, European Center for Disease Prevention and Control; EFSA, European Food Safety Authority; DEFRA, Department for Environment, Food and Rural Affairs. Databases/Platforms include GDW, LIMS, EPIS, RASFF, and Enterobase.

Presumptive *Salmonella* isolates are submitted by frontline testing laboratories to the *Salmonella* Reference Service for confirmation and further characterization (Figure 1). On receipt the DNA is extracted using the Qiasymphony automated DNA extraction machine [Qiagen, UK] and sequenced using the Illumina HiSeq 2500 platform in rapid run mode (2 × 100 bp reads). The samples are batched with other pathogen isolates received for sequencing for the maximum capacity of 96 isolates per lane, per flowcell. The quality of raw FASTQ files is evaluated using an in-house program, qa_and_trim, which determines the metric yield of the sample (where yields of data from an isolate are below 150 Mb and are repeated) and trims the files using Trimmomatic (14) (using the parameters LEADING:30, TRAILING:30, SLIDINGWINDOW:10:20, and MINLEN:50). All subsequent analysis is carried out on the trimmed files. As previously described, the PHE KmerID pipeline (https://github.com/phe-bioinformatics/kmerid) is used to compare the sequenced reads with published genomes to identify the bacterial species and *Salmonella* subspecies (13). The quality of the sample is further evaluated by MLST using the Achtman seven gene scheme (15) (MOST, https://github.com/phe-bioinformatics/MOST) (16). Each sample is assigned a “traffic light” color depending on its coverage metrics: *Green*-maximum percentage non-consensus depth <15%, minimum consensus depth >2, percentage coverage = 100%, and that the ST determination has not failed; *amber*-maximum non-percentage consensus depth is ≥15% or minimum consensus depth is between 0 and 2 (inclusive); *red*-percentage coverage <100% or the ST determination has failed.

*Salmonella* serovar determination is predicted based on the *Salmonella* eBURST group (eBG) or Sequence Type (ST) (15) and checked against a validated PHE database (13). Validation of eBG and ST for inferring serovar is an ongoing process and currently requires a minimum of three isolates within that group to have been validated with the SeqSero profile (17) and confirmed with full phenotypic serology of both the somatic and flagella antigens (8, 9). Partial phenotypic serology is also currently performed when STs contain more than one serovar (polymorphic) or where referring primary diagnostic laboratories refer mixed cultures or they indicate conflicting serology results on the request form. To ensure reports are kept within TAT, where there are novel STs, the isolate is assigned an internal temporary ST until it has been submitted to a public repository and assigned a standard ST. The temporary ST is then overwritten with the new ST.

Microbial fine typing is achieved by utilizing the high discriminatory power of single nucleotide polymorphisms (SNP). A bioinformatics application, SnapperDB has been developed to quantify SNP relatedness and derive an isolate level nomenclature termed the “SNP Address” (18). This applies multi-threshold single linkage clustering to describe an isolate's position in the population structure of a given Salmonella eBG. Single-linkage clustering is performed at seven descending thresholds of SNP distance; 250, 100, 50, 25, 10, 5, and 0. This clustering results in a discrete seven-digit code where each number represents the cluster membership at each descending SNP distance threshold. Maximum likelihood phylogenies of selected strains of interest are constructed based on SNPs extracted from SnapperDB using RaxML v8.2.8 (19).

Turnaround times (TATs) before WGS averaged around 20 days from isolate receipt to reporting of validated results; Biochemistry−5–28 days, Serotyping−3–21 days, PT−3–10 days, PFGE−7–10 days. The average TAT for results utilizing WGS is now 10 days but these reports can be issued in as little as 6 days and can replace all of the previous methods. The reduced TAT and improvement of laboratory typing data has improved the outbreak investigation process since data is received quicker for analysis and case definitions have been refined and based on the enhanced granularity of the typing. The validation process for reporting laboratory results has remained the same with a two stage process involving the technical and medical validator checking the validity and quality metrics (such as the yield) of the WGS data and other performed tests for *Salmonella* identification. Participation in External Quality Assessment (EQA) schemes remain the same with the addition of specific EQAs now in place for cluster detection via genomic methods.

### Antimicrobial Resistance and Clinical Interpretation

Using WGS data, genetic antimicrobial resistance (AMR) determinants are sought using reference mapping approaches as previously described (20, 21). Resistance genes are identified by comparison to an in-house curated library collated from publicly accessible databases (PRJNA313047) (22, 23). Known chromosomal mutations, acquired resistance genes and resistance-conferring mutations relevant to β-lactams (including carbapenems), fluoroquinolones, aminoglycosides, chloramphenicol, macrolides, sulphonamides, tetracyclines, trimethoprim, and fosfomycin and acquired genes associated with colistin resistance are included in the reference database. Genotypic markers to infer phenotypic antimicrobial resistance have been recently validated (20, 21) but further work is required to translate this into a clinically useful format (24). Phenotypic antimicrobial sensitivity testing (AST) are carried out to provide minimal inhibitory concentrations (MICs) (according to EUCAST guidelines http://www.eucast.org/clinical_breakpoints/). These are provided for clinical management where requested by diagnostic laboratories and a percentage of *Salmonella* are routinely phenotypically tested to check clinically important (e.g., bacteraemia or treatment failure cases) isolates and for horizon scanning purposes to detect novel and /or emerging mechanisms of resistance.

### Reporting Results and Integrated Analysis of the Data

Frontline diagnostic laboratories report the isolation of *Salmonella* spp to PHE via the Second Generation Surveillance System (SGSS), a database that stores and manages data on laboratory isolates and results, and is the preferred method for capturing routine laboratory surveillance data on all infectious diseases and antimicrobial resistance from laboratories across England (25). This data is used for the monitoring of the overall number of *Salmonella* isolated at frontline laboratories and the number of isolates referred to GBRU. WGS results (ST, eBG, serovar, and SNP address) populate a Laboratory Information Management System (LIMS) at the *Salmonella* reference laboratory, where they are validated and reported to the sending clinician (Figure 1). The WGS data are currently only available via a restricted access web-based system, the Gastro Data Warehouse (GDW), a secure, encrypted, rationalized database containing results on all isolates processed by GBRU (Figure 1). PHE staff access data for cases within their region(s) on GDW via a web-enabled interface through which line-listings of case epidemiological data and sequencing results can be extracted based on case demographic and/or sequencing results, such as inferred serovar, ST, or SNP address. GDW also contains a cluster extraction functionality which allows users to search for SNP clusters based on desired temporal, size, and SNP distance level thresholds. This allows real-time surveillance of microbiological clusters by regional and national teams in line with the TAT stated above.

Routine surveillance and monitoring of *Salmonella* trends for general surveillance and risk assessment purposes is still carried out at the serovar level. SNP typing is routinely undertaken for the most commonly reported eBGs, and new eBGs/STs can be added to the routine pipeline as necessary; currently 86% of isolates received undergo SNP typing in real time. For those eBG not subject to SNP typing, the exceedance algorithm applied on the SGSS data is still used for outbreak detection at the serovar level (26). Where a potential outbreak event is detected, retrospective SNP typing of all the isolates within the ST/eBG is undertaken to refine outbreak detection and prospective SNP typing becomes routine. The SNP address is now utilized by PHE epidemiologists and microbiologists as the primary method for identifying microbiological clusters of gastrointestinal infections in England to detect potential outbreak events. Case isolates that fall within a 5-SNP single linkage cluster are considered likely to be exposed to a common source of contamination. The number of SNPs within a 5-SNP linkage cluster will vary depending on the size, type, source, and length of the outbreak. For example an international outbreak of *S*. Enteritidis, spanning over 3 years, had two distinct 5-SNP single linkage clusters even though they were from the same source of eggs from Poland. Cluster 1 had a maximum SNP distance of 18 SNPs whereas Cluster 2 had 37 SNPs (27). Validation studies (28) and prospective use in outbreak investigations (29, 30) indicate that the 5-SNP level is suitable for detection of salmonellosis cases that are likely to be epidemiologically linked and share a common exposure or source of infection.

In order to analyze and act on the data in real time in a systematic manner and manage the high volume of data generated by WGS, an automated reporting system, the “SNP Cluster Tool,” has been developed using the statistical software R (31). The tool identifies and extracts epidemiological and sequencing data for clusters of two or more cases which cluster at the 5-SNP level where at least one case has been reported in the preceding week. Clusters are automatically summarized by rule-based categories in terms of case demographics (age, sex, geographic distribution, and travel history) and cluster-level characteristics (size, period of time since the first case was reported and cluster growth rate). The resultant summary tables are distributed on a weekly basis to microbiologists and epidemiologists working on *Salmonella* surveillance at the national and at the regional level. This automated approach facilitates rapid cluster assessment and prioritization of clusters requiring further investigation. The 5-SNP level is used primarily as an initial cluster extraction and assessment threshold but subsequent analysis of the cluster epidemiology and phylogeny may result in this threshold being extended as guided by the epidemiology. Where warranted this may even lead to the subsequent selection of more than one epidemiologically or phylogenetically related 5-SNP cluster to define the case definition for an outbreak investigation (29, 32). A key difference in defining SNP-clusters both microbiologically and epidemiologically compared to previous typing methods and epidemiological approaches is that the microbiological characterization is considered sufficiently discriminatory that clusters are usually defined independently of time. Therefore, in most national outbreaks we apply non time-limited, phylogeny-based case definitions and, in addition, no longer apply some traditional exclusion criteria such as travel history.

Phylogenetic trees are generated for clusters which have been prioritized for further assessment. Phylogenetic analysis provides insight into the genetic relationship between outbreak isolates which may reveal underlying epidemiological processes or sampling dynamics (33). In addition, phylogenetic context determined through assessing available epidemiological data for isolates related at a wider genetic threshold may assist hypothesis generation may assist hypothesis generation in terms of geographical origin or potential source. Phylodynamic reconstruction using Bayesian evolutionary analysis (34) may also be deployed in outbreak settings to estimate the temporal origin of the outbreak strain and to identify changes in population size over time. These approaches can be particularly valuable for outbreaks with long durations and where the assessment of the success of interventions is needed (27).

**Figure 2:**
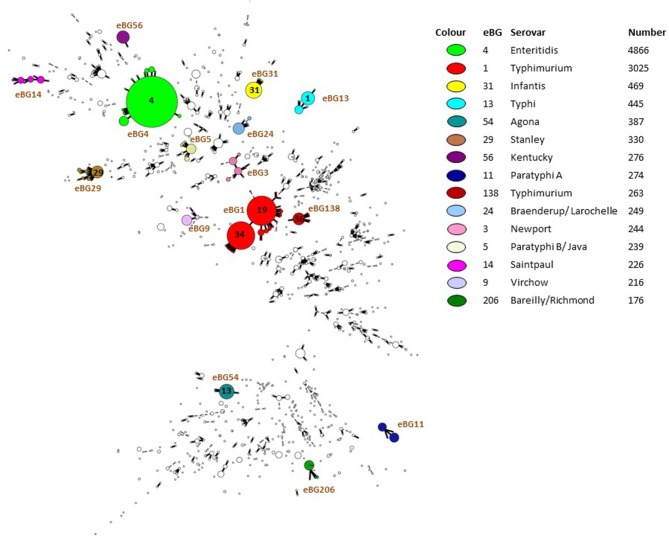
Population structure of 16,854 *Salmonella* isolated from humans and submitted to PHE from local and regional hospital laboratories in England and Wales between April 2016 and March 2018.

PHE also make validated FASTQ sequences publically available (Figure 1) by routinely uploading *Salmonella* sequence data to NCBI BioProject PRJNA248792 (https://www.ncbi.nlm.nih.gov/bioproject/?term=PRJNA248792). Basic metadata is provided including the Month/Year, Country, Isolation source (e.g., human, animal, food), serovar and ST. As of 20th March 2019, 45,413 SRA experiments are available for analysis. Data from NCBI is routinely imported to Enterobase, so that other organizations can utilize its online tools such as analyzing population structures (Figure 2) or utilizing cgMLST tools and compare PHE genomes with their own data in outbreak detection. This enables any user to have access to the data for comparison analysis and has enabled real-time comparison of outbreaks at the international level.

### Experiences and Outputs of WGS Implementation at PHE 2016–2018

**Figure 3:**
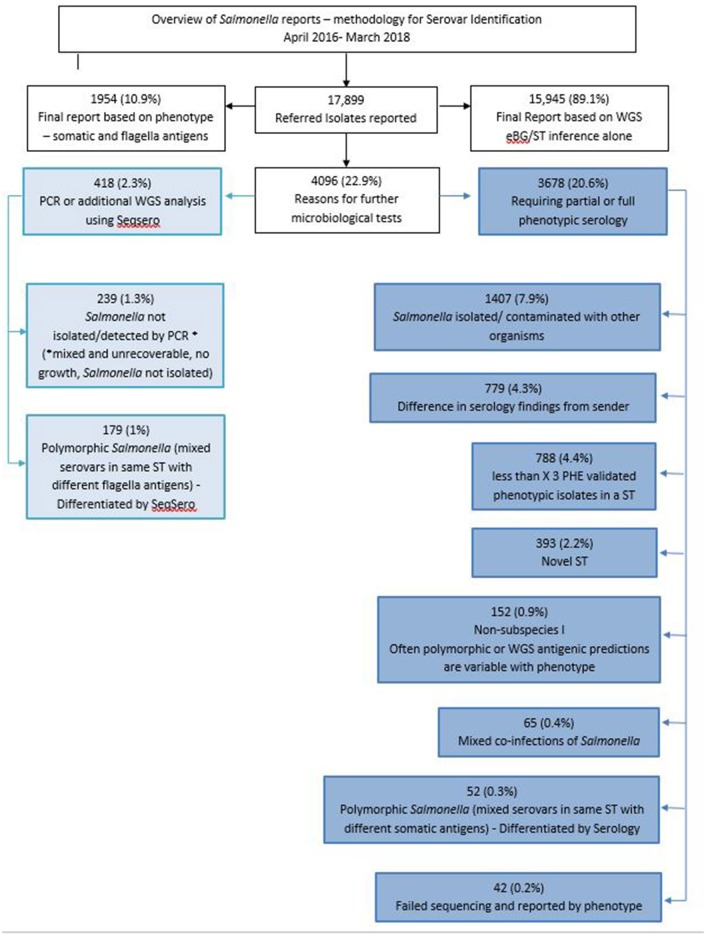
Overview of *Salmonella* reports and methodology for serovar identification, April 2016–March 2018.

WGS has not yet fully replaced traditional typing methods, a review of the 17,899 confirmed *Salmonella* laboratory results reported between April 2016 and March 2018 indicated that 89.1% of *Salmonella* serovars were reported by eBG/ST inference alone while the other 10.9% were reported on the antigenic phenotype (Figure 3).

Of the 17,899 reports, a total of 4,096 (22.8%) isolates required further microbiological tests including serology and PCR (Figure 3). The main reasons for additional serological testing included novel STs, mixed cultures referred by the sending laboratory and polymorphic *Salmonella* (more than one serovar within a ST) (Figure 3).

Out of the 17,899 isolates reported between April 2016- March 2018, 2,128 (11.8%) were tested phenotypically for AST (Table 1). There were no resistant *Salmonella* detected using phenotypic methods that were missed using WGS surveillance during this period, although results continue to show that genotypic AMR mutations do not always express phenotypically (20, 21). The use of WGS has enabled real-time, high throughput, routine surveillance of resistance determinants to detect emerging threats, such as the confirmation of the first ESBL *S*. Typhi case in the UK (35). A useful benefit of genotypic characterization of AMR determinants is the ability to rapidly add additional gene targets to the database, enabling rapid screening of thousands of isolates in a short period of time. In 2015, PHE demonstrated the use of WGS for rapid screening of the genomes of ~24,000 *Salmonella enterica, E. coli, Klebsiella* spp., *Enterobacter* spp., *Campylobacter* spp. and *Shigella* spp. to identify novel transmissible colistin resistance (mcr-1) in 15 human and food isolates (36). Another example of utilizing WGS AMR data has been monitoring of emerging resistance to a first-line antibiotic azithromycin in *Salmonella* spp (37).

**Table 1:** Current criteria for selection of *Salmonella* isolates for phenotypic antimicrobial sensitivity testing by in-agar dilution.

**Criteria**	**No. isolates tested 1 April 2016–31 March 2018**
All *S*. Typhi isolates	457
All *S*. Paratyphi A isolates	284
All *S*. Paratyphi B isolates	36
All *S*. Paratyphi C/Choleraesuis and variants of 6,7:C:1,5 isolates	6
Non-typhoidal *Salmonella* (NTS) Bacteremia's	433
Invasive or complex NTS clinical cases (from patient sources other than feces and blood and by request).	103
Food, animal and environmental *Salmonella* isolates	161
From analysis of *Salmonella* sequencing data: all isolates that genotypically show the presence of one or more extended spectrum ß-lactamase genes	200
From analysis of *Salmonella* sequencing data: all isolates that genotypically show the presence of one or more extended spectrum ß-lactamase genes	163
From analysis of *Salmonella* sequencing data: all isolates that genotypically show the presence of one or more Carbapenamase resistance genes	1
From analysis of *Salmonella* sequencing data: all isolates that genotypically show the presence of two or more macrolide resistance genes	240
From analysis of *Salmonella* sequencing data: all isolates that genotypically show the presence of one or more colistin resistance genes	45
Total No. Isolates	2,128

*Table summarizing the current criteria for phenotypic testing of isolates for antimicrobial resistance testing (AST) and the numbers tested for AST of the 17,899 isolates reported between April 2016 and March 2018. Note, not all resistance gene markers will express phenotypically. Numbers are the total tested, not necessarily the number with antimicrobial resistance*.

Since implementing WGS methods in April 2014, *Salmonella* reporting trends in England and Wales have been generally consistent with previous years. However, assessing laboratory data using eBG rather than serovar has shown that analysis of the data at the serovar level doesn't optimally reflect the incidence of genetically related groups. Assessment of eBGs reported between April 2016 and March 2018 shows that eBG 4 (*S*. Enteritidis, 4,866 isolates), eBG 1 (*S*. Typhimurium, 3,025 isolates) and eBG31 (*S*. Infantis,469 isolates) constitute the main burden of salmonellosis in England and Wales (Figure 2) as also reflected in analysis at the serovar level (5,240, 3,649, and 540 serovar reports, respectively). However, for polyphyletic serovars (serovars found in multiple eBGs), for example S. Newport, “rank” in terms of number of reports varies substantially when comparing the traditional serovar (671 isolates) to the multiple eBGs of which it is comprised. *S*. Newport was the third most commonly reported serovar between April 2016 and March 2018, however is comprised of multiple eBGs (eBG 2,3,7,35), with the most commonly reported *S*. Newport eBG (eBG3) being the 14th most commonly reported eBG (244 isolates) overall (Figure 2).

Of the 17,899 isolates reported from April 2016 to March 2018, 13,948 *Salmonella* isolates clustered with at least one other isolate at the 5-SNP level. These formed 2,007 clusters, distributed across 46 eBGs (Table 2). This time period was selected to identify the number of active clusters (i.e., the number of clusters with at least one new case added), however cluster statistics were analyzed using all cases with membership in the cluster regardless of when the result was reported. The majority of reported clusters were small, with only 29% of clusters constituting five or more cases (range: 2–423 cases, median: 3 cases). When these clusters were analyzed including all cases in the 5-SNP cluster, including those prior to March 2018, fifty-eight percent of clusters contained cases reported over a period of time exceeding 3 months (range: 0.03–115 months [linked to historical cases in these clusters], median: 6 months). Clusters of eBG4 (*S*. Enteritidis) constitute the majority of the longest duration clusters, and there is evidence gained from retrospective sequencing and analysis of isolates from 2008 to 2015 that an outbreak linked to feeder mice has persisted have persisted for over 10 years to date (38).

**Table 2:** Characteristics of *Salmonella* WGS clusters, England, April 2016–March 2018.

eBG*	**Serovar**	**Clusters**	**Cluster size**			**Cluster duration (months)**			**Cluster cases per week**
			**Median**	**Min**	**Max**	**Median**	**Min**	**Max**	**Max**
4	Enteritidis	616	3.0	2	423	10.00	0.03	115.00	9.0
1	Typhimurium	606	3.0	2	165	4.00	0.03	74.00	5.0
31	Infantis	75	2.0	2	61	4.00	0.03	41.00	3.0
13	Typhi	67	3.0	2	112	12.00	0.10	72.00	2.0
11	Paratyphi A	59	3.0	2	36	15.00	0.13	64.00	2.0
29	Stanley	52	2.0	2	9	3.00	0.03	39.00	3.0
54	Agona	51	2.0	1	72	5.00	0.03	89.00	2.0
5	Java	45	2.0	2	13	5.00	0.03	47.00	2.0
56	Kentucky	37	2.0	2	28	8.00	0.03	40.00	2.0
9	Virchow	35	2.0	2	38	13.00	0.07	46.00	2.0
22	Hadar	34	2.5	2	17	5.00	0.07	41.00	3.0
138	Typhimurium	34	2.0	2	8	0.73	0.03	28.00	3.5
24	Braenderup	30	2.5	2	69	7.00	0.03	49.00	3.5
206	Bareilly	30	2.0	2	27	5.00	0.03	39.00	2.0
3	Newport	25	2.0	2	20	2.00	0.03	38.00	2.0
7	Newport	22	2.5	2	16	1.50	0.03	34.00	2.0
34	Bovis morbificans	18	2.5	2	24	6.00	0.10	38.00	2.5
62	Mbandaka	17	2.0	2	5	7.00	0.03	35.00	2.0
247	Mikawasima	16	3.0	2	16	1.00	0.16	24.00	4.0
44	Oranienburg	13	4.0	2	19	15.00	0.49	43.00	1.0
49	Chester	13	3.0	2	32	19.00	0.03	42.00	2.0
2	Newport	12	3.0	2	45	7.00	0.03	42.00	2.0
35	Newport	10	2.0	2	5	3.00	0.20	27.00	1.5
41	Oranienburg	10	3.0	2	29	2.00	0.03	17.00	2.0
65	Anatum	9	2.0	2	3	0.72	0.03	15.00	2.0
205	Weltevreden	7	2.0	2	7	0.66	0.03	13.00	2.0
12	Brandenburg	6	3.0	2	5	4.00	0.16	10.00	2.0
64	Kottbus	6	2.5	2	7	0.64	0.20	12.00	1.5
17	Javiana	5	3.0	2	4	7.00	0.03	35.00	2.0
61	Litchfield	5	2.0	2	4	5.00	1.00	37.00	1.0
70	Virchow	5	3.0	2	7	1.00	0.10	2.00	2.0
164	Kentucky	5	2.0	2	3	12.00	0.16	29.00	1.0
26	Heidelberg	4	2.0	2	3	0.71	0.03	3.00	2.0
32	Java	4	2.0	2	3	0.29	0.03	2.00	1.0
67	Give	4	9.0	2	17	10.00	0.03	17.00	1.5
271	Indiana	4	7.0	2	28	9.00	0.03	17.00	2.0
291	Kedougou	4	3.0	2	50	15.50	0.36	27.00	2.0
421	Adjame	3	5.0	4	7	0.69	0.23	1.00	1.0
270	Liverpool	2	4.0	3	5	6.34	0.69	12.00	2.0
292	Agbeni	2	3.5	2	5	10.00	1.00	19.00	1.0
57	Derby	1	2.0	2	2	12.00	12.00	12.00	1.0
244	Derby	1	20.0	20	20	40.00	40.00	40.00	1.0
264	Derby	1	5.0	5	5	30.00	30.00	30.00	1.0
1483	Abony	1	4.0	4	4	28.00	28.00	28.00	1.0
1992	Carno	1	12.0	12	12	5.00	5.00	5.00	1.0

Table describing the characteristics of Salmonella whole sequencing genome clusters in order of decreasing cluster burden by eBG (*where the eBG is not defined, the ST will be specified). Each eBG is characterized in terms of the number of clusters detected during the surveillance period (with the data for each cluster including isolates falling into the clusters outside the study period), the number of cases within clusters, the age of the cluster in months and the number of cases detected per cluster per week. The maximum cluster duration will date back to any historical strains that have been sequenced and fall into the cluster.

## Discussion

### Improvement in Reference Services Including Diagnostics

Implementation of WGS has transformed reference microbiology services both in terms of improved accuracy of results (13), and reduced turnaround times by ~50%. Further reduction of TATs is possible but we are currently limited by the requirement to batch process samples and the continuation of additional phenotypic work. As routine WGS is implemented for more organisms across PHE, the increase in numbers will enable increased sequencing runs and hence a reduction in TATs. The simplification of sample processing also reduces the potential for laboratory errors and minimizes staff exposure to pathogens thereby improving safety practices. In addition, we have utilized the sequence data generated through routine testing to develop specific, rapid real-time PCR tests to assist in the management of patients including for the rapid differential diagnoses of typhoidal from non-typhoidal *Salmonella* (39) and to detect azithromycin resistant infections (in house assay). This has had a direct clinical impact as same day testing can be provided for urgent clinical cases. It is also worth noting the rapidly developing technology of desktop and nanopore sequencing becoming available to clinical laboratories. As these technologies become more affordable and common in clinical practice, real-time diagnostic sequencing will be able to identify pathogens, detect virulence factors and drug resistance markers to support clinical treatment. Currently local laboratories are legally required to notify PHE of the isolation of *Salmonella* sp. from a human sample; although further characterization is not mandated in the current legislation (4, 5). Fortunately, the majority (>95%) of isolated Salmonellae are currently sent to the reference laboratory for further typing to enable a robust national surveillance system. A move to sequencing occurring locally could pose a risk to a cohesive, representative national data set due to the lack of legal basis for such, though we think it likely that a system for sequence sharing would be set up to address this. However, even with the implementation of PCR which has been in place for over a decade, not all frontline laboratories use this technology. Benchtop sequencing is unlikely to have a large impact on the current reference services model in the short term with the current infrastructure in place.

### Enhanced Surveillance and Outbreak Investigation

Although published evidence does not yet support the use of WGS-inferred antimicrobial susceptibility to guide clinical management of individual cases (24), studies have shown WGS to be an extremely rapid, robust, accurate tool for AMR surveillance in food-borne pathogens such as *Salmonella* spp. (20, 21). It is expected that information derived from WGS-based studies will increasingly be used to inform public health interventions aimed at limiting further dissemination of AMR genes in foodborne pathogens.

Considering the variability in eBG for some serovars (Figure 2), assessing *Salmonella* trends by eBGs, where available, may be more appropriate than by serovar, as differentiation by serovar does not optimally define the population heterogeneity to the level possible using eBG. Therefore, we are moving more to the use of eBG and in future eBG/ST for general surveillance, trend monitoring and outbreak detection based on exceedance algorithms. This work is still underway to integrate into routine surveillance systems.

The high-resolution typing provided by WGS for routine surveillance is facilitating the improved detection of smaller and geographically widespread clusters of common serovars such as *S*. Enteritidis and—especially for common strains. In these cases, the detection of a national outbreak would not have been possible without the use of WGS to delineate the outbreak strain from background numbers of commonly reported serovars/serovar and phage type combinations, and WGS can provide a much more refined case definition (38). Previous methods such as PT did not provide information on genotypic relationships and with common PTs, outbreak strains may have been overlooked particularly with ongoing outbreaks involving multiple PTs. In addition, cases have been epidemiologically investigated that were not genetically linked to the outbreak strain (38). Although, PFGE and PulseNet has been the backbone in the detection and sharing of outbreaks (https://www.cdc.gov/pulsenet/pathogens/pfge.html) on a global scale, there have been occasions where PFGE has not always been useful in detecting the same clone (40). The introduction of WGS in PHE and other agencies has enhanced the way we compare outbreak isolates and has facilitated an understanding of sources of outbreaks that would not have been possible with previous typing methods (30, 32, 33).

### Data Accessibility and Integration of Cross Disciplinary Working

Key to the integration of epidemiology and phylogenetic information at PHE is data management and real time accessibility via the GDW database (Figure 1), as well as the SNP address nomenclature. The use of WGS generates a huge volume of data that requires further assessment by epidemiologists to determine if there is a need for action/outbreak investigation. The large amount of sequencing data generated for analysis each week necessitated the development of automated data extraction and analysis tools that have the capacity to deal with large amounts of data to aid rapid assessment and prioritization for further investigation. The sharing of the summary outputs of clusters and access to the WGS results integrated with basic case epidemiological data in a single database accessible by microbiologists, bioinformaticians and epidemiologists at the local, regional and national level means that local, regional and national teams are able to interpret fine typing microbiological data together with epidemiological data as part of routine surveillance, and target their investigations/resources where cases are most likely linked to a common source of contamination. A welcome consequence of implementing WGS has been closer working between public health infectious disease experts resulting in an enhanced, multidisciplinary approach to GI surveillance and outbreak investigation (Figure 1).

Inter-agency sharing and comparisons of microbiological, epidemiological, and food chain analysis results is necessary for effective food safety and control of zoonotic diseases at the UK and at the international level. The comparison of WGS results enhances effective assessment of cross-border threats and participation in multi-country outbreak investigations. Sharing raw sequence data, along with utilizing international information platforms supported by European Center for Disease Prevention and Control (ECDC) for the sharing of microbiological and epidemiological information, has proved successful for collaborative multi-agency, multi-country outbreak investigations (32, 33, 41).

### Gaps, Limitations, and Future Work

As with any new system, there are limitations and there is room for improvement. A robust microbiological surveillance system depends upon high isolate referral rates, so, while there is currently high coverage for human diagnostic samples, there are laboratories (particularly in the private sector) that do not refer food isolates for further characterization. Consequently, crucial information from the food chain that could help inform hypothesis generation and target outbreak investigation and food chain analysis is being missed. Currently there is no system in place for routine sharing of animal data outside of outbreak investigations but PHE are addressing this together with the Animal and Plant Health Agency (APHA). In addition, the potential move to culture-independent diagnostic tests for GI pathogens by hospital laboratories threatens to reduce the representativeness of WGS data as isolates would not always available for sequencing.

Although a small number of isolates are still being fully phenotypically serotyped due to validation of novel STs (Figure 3), *in silico* serotyping methods such as SeqSero (17) or SISTR (42) hold great promise in providing a direct replacement for prediction of individual somatic and flagella antigens, as currently defined by the Kaufmann-White-Le Minor scheme. It should be noted however that genotypic prediction does not always correlate to phenotypic expression which is problematic for defining novel *Salmonella* strains. We recognize that continuing to perform phenotypic serology routinely is not desirable or sustainable and we aim to cease all traditional serotyping methods in future.

Additional limitations include the necessity of pure cultures required for DNA extraction as contamination will interfere with bioinformatic outputs including accurate sequence typing, fine typing results of SNP analysis and correct calling of AMR gene determinants. Batch processing of samples is still required for sequencing to improve efficiency and maintain cost-effective operations; as a result, TATs are typically in excess of 7 days and in urgent typhoidal cases, PCR (39) is still required to provide a preliminary identification.

Recent publications (20, 21) have demonstrated the utility of WGS-inferred antimicrobial susceptibility for clinical management, rapid surveillance initiatives and monitoring of emerging resistance. It is acknowledged that novel mechanisms of resistance could be missed using genotypic determination of AMR and how the presence of AMR determinants relates to MICs is as yet still not fully understood, therefore a certain level of phenotypic testing is still required. MIC prediction by WGS and machine learning is currently being investigated (43), where the observed MIC is underpinned by genetic factors encoded in the DNA, prediction should be possible and a potential model for the future. It is crucial to perform active curation of the resistance gene databases to maintain the high sensitivity of genotypic prediction especially due to novel, emerging resistance mechanisms. Our in-house pipeline, for instance, does not detect impermeability or efflux pumps as these mechanisms are not always encoded by a single gene that can be easily detected.

The SNP address derived from the PHE pipeline has been utilized to identify microbiologically linked cases through collaborative working and sharing of sequence data in international outbreak investigations. However, there are multiple different pipelines and nomenclatures used in different organizations, so WGS results may not always be easily communicated between agencies using different systems in the initial stages of detection and assessment of threats. Real-time multi-country comparison of WGS data remains challenging, and the future use of harmonized typing schemes and supporting infrastructure is welcomed (44, 45) and validation studies have already begun (46). One example is the NCBI Pathogen Detection Portal (https://www.ncbi.nlm.nih.gov/pathogens) and is a working example of close to real-time comparison system for surveillance of bacterial pathogens using WGS. There are multiple caveats, such as making the data public and being able to interpret phylogenetic trees but this approach does work and an open framework for all to access.

The high volume of clusters detected each week and longevity of some clusters due to persistent sources of contamination can be challenging in terms of consistent resource allocation. A high-level of expertise is required to interpret WGS data in combination with epidemiological evidence.

## Conclusion

### The Whole Is More Than the Sum of Its Parts

The integration of routine WGS as a replacement for traditional microbiological methods has revolutionized reference microbiology and impacted real-time surveillance of gastrointestinal pathogens for improved public health outcomes. PHE have now implemented routine WGS methods for *Salmonella* (13)*, Shigella* (47, 48)*, Campylobacter, Escherichia* (48, 49), *Listeria* (50)*, Vibrio* (51), and *Yersinia* species (52). It is envisioned that WGS methods will be implemented for all gastrointestinal bacterial pathogens services at PHE within the next few years.

The large volume of data generated by the use of WGS has required additional tools be developed to facilitate surveillance, cluster assessment and prioritization, and outbreak detection; using these tools these processes have become more discriminatory and can occur in near real-time compared to previous typing methodologies. This has improved outbreak detection, hypothesis generation, and source attribution in ways not previously possible.

The posting of sequences on a publicly accessible database means other countries can compare with their in-house databases and has facilitated substantial international collaboration that would not have possible if all data was only kept in-house.

International harmonization of WGS typing methods for surveillance is crucial and still in the development phase. Close collaboration between epidemiologists, bioinformaticians, microbiologists, clinicians and food safety experts is essential to maximize the public health potential provided by WGS.

## Data Availability Statement

All datasets generated for this study are included in the article. In addition, raw sequence data described in this article is publically available on NCBI, PHE Salmonella Bioproject: PRJNA248792.

## Author Contributions

MC, SN, and MD implemented the wet lab WGS pipelines, performed analysis, and identification. MD, MC, and GG performed AST identification and reporting. TD and HH performed bioinformatic analysis. LL, JM, AM, and RS performed cluster analysis and epidemiological investigations. DP performed data analysis. MC and KG wrote the manuscript. TD, LL, SN, JM, AM, HH, GG, DP, MD, and RS contributed to the manuscript.

### Conflict of Interest

The authors declare that the research was conducted in the absence of any commercial or financial relationships that could be construed as a potential conflict of interest.

## References

[1] Taylor AJ Lappi V Wolfgang WJ Lapierre P Palumbo MJ Medus C. Characterization of foodborne outbreaks of *Salmonella enterica* serovar enteritidis with whole-genome sequencing single nucleotide polymorphism-based analysis for surveillance and outbreak detection. J Clin Microbiol. (2015) 53:3334–40. 10.1128/JCM.01280-15 26269623 PMC4572550

[2] Wuyts V Denayer S Roosens NH Mattheus W Bertrand S Marchal K. Whole genome sequence analysis of *Salmonella* Enteritidis PT4 outbreaks from a national reference laboratory's viewpoint. PLoS Curr. (2015) 7:1–14. 10.1371/currents.outbreaks.aa5372d90826e6cb0136ff66bb7a62fc 26468422 PMC4593640

[3] Thomas M Fenske GJ Antony L Ghimire S Welsh R Ramachandran A. Whole genome sequencing-based detection of antimicrobial resistance and virulence in non-typhoidal *Salmonella enterica* isolated from wildlife. Gut Pathog. (2017) 9:66. 10.1186/s13099-017-0213-x 29201148 PMC5697165

[4] England PH The Health Protection (Notification) Regulations 2010. Public Health England, The Stationery Office Limited (2010). p. 659.

[5] Wales PH The Health Protection (Notification) (Wales) Regulations 2010. Public Health England, The Stationery Office Limited (2010). p. 1546.

[6] Cowan S Steel T Barrow GI Feltham RKA Cowan and Steel's Manual for the Identification of Medical Bacteria. Cambridge: Cambridge University Press (1993).

[7] Hopkins KL Peters TM Lawson AJ Owen RJ. Rapid identification of *Salmonella enterica* subsp. arizonae and *S enterica* subsp diarizonae by real-time polymerase chain reaction. Diagn Microbiol Infect Dis. (2009) 64:452–4. 10.1016/j.diagmicrobio.2009.03.022 19631101

[8] Grimont PADWFX Antigentic Formulae of the Salmonella Serovars. Institut Pasteur: WHO Collaborating Centre for Reference and Research on Salmonella (2008).

[9] Guibourdenche M Roggentin P Mikoleit M Fields PI Bockemuhl J Grimont PA. Supplement 2003-2007 (No. 47) to the White-Kauffmann-Le Minor scheme. Res Microbiol. (2010) 161:26–29. 10.1016/j.resmic.2009.10.002 19840847

[10] Callow BR. A new phage-typing scheme for *Salmonella* Typhi-murium. J Hyg. (1959) 57:346–59. 10.1017/S0022172400020209 13807011 PMC2218121

[11] Peters TM. Pulsed-field gel electrophoresis for molecular epidemiology of food pathogens. Methods Mol Biol. (2009) 551:59–70. 10.1007/978-1-60327-999-4_6 19521867

[12] Hopkins KL Peters TM de Pinna E Wain J. Standardisation of multilocus variable-number tandem-repeat analysis (MLVA) for subtyping of *Salmonella enterica* serovar Enteritidis. Euro Surveill. (2011) 16:19942. 10.2807/ese.16.32.19942-en 21871223

[13] Ashton PM Nair S Peters TM Bale JA Powell DG Painset A. Identification of *Salmonella* for public health surveillance using whole genome sequencing. PeerJ. (2016) 4:e1752. 10.7717/peerj.1752 27069781 PMC4824889

[14] Bolger AM Lohse M Usadel B. Trimmomatic: a flexible trimmer for Illumina sequence data. Bioinformatics. (2014) 30:2114–20. 10.1093/bioinformatics/btu170 24695404 PMC4103590

[15] Achtman M Wain J Weill FX Nair S Zhou Z Sangal V. Multilocus sequence typing as a replacement for serotyping in *Salmonella enterica*. PLoS Pathog. (2012) 8:e1002776. 10.1371/journal.ppat.1002776 22737074 PMC3380943

[16] Tewolde R Dallman T Schaefer U Sheppard CL Ashton P Pichon B. MOST: a modified MLST typing tool based on short read sequencing. PeerJ. (2016) 4:e2308. 10.7717/peerj.2308 27602279 PMC4991843

[17] Zhang S Yin Y Jones MB Zhang Z Deatherage Kaiser BL Dinsmore BA. *Salmonella* serotype determination utilizing high-throughput genome sequencing data. J Clin Microbiol. (2015) 53:1685–92. 10.1128/JCM.00323-15 25762776 PMC4400759

[18] Dallman T Ashton P Schafer U Jironkin A Painset A Shaaban S. SnapperDB: a database solution for routine sequencing analysis of bacterial isolates. Bioinformatics. (2018) 34:3028–29. 10.1093/bioinformatics/bty212 29659710

[19] Stamatakis A. RAxML version 8: a tool for phylogenetic analysis and post-analysis of large phylogenies. Bioinformatics. (2014) 30:1312–3. 10.1093/bioinformatics/btu033 24451623 PMC3998144

[20] Day MR Doumith M Do Nascimento V Nair S Ashton PM Jenkins C. Comparison of phenotypic and WGS-derived antimicrobial resistance profiles of *Salmonella* enterica serovars Typhi and Paratyphi. J Antimicrob Chemother. (2017) 73:365–72. 10.1093/jac/dkx379 29216342

[21] Neuert S Nair S Day MR Ashton PM Mellor KC Jenkins C. Prediction of phenotypic antimicrobial resistance profiles from whole genome sequences of non-typhoidal *Salmonella* enterica. Front Microbiol. (2018) 9:592. 10.3389/fmicb.2018.00592 29636749 PMC5880904

[22] Zankari E Hasman H Cosentino S Vestergaard M Rasmussen S Lund O. Identification of acquired antimicrobial resistance genes. J Antimicrob Chemother. (2012) 67:2640–4. 10.1093/jac/dks261 22782487 PMC3468078

[23] Orlek A Phan H Sheppard AE Doumith M Ellington M Peto T. A curated dataset of complete *Enterobacteriaceae* plasmids compiled from the NCBI nucleotide database. Data Brief. (2017) 12:423–6. 10.1016/j.dib.2017.04.024 28516137 PMC5426034

[24] Ellington MJ Ekelund O Aarestrup FM Canton R Doumith M Giske C. The role of whole genome sequencing in antimicrobial susceptibility testing of bacteria: report from the EUCAST Subcommittee. Clin Microbiol Infect. (2017) 23:2–22. 10.1016/j.cmi.2016.11.012 27890457

[25] England PH Laboratory Reporting to Public Health England: A Guide for Diagnostic Laboratories. PHE publications gateway number: 2016137. London: Public Health England (2016).

[26] Noufaily A Farrington P Garthwaite P Enki DG Andrews N Charlett A Detection of Infectious Disease Outbreaks From Laboratory Data With Reporting Delays. Open University (2016).

[27] Pijnacker R Dallman TJ Tijsma ASL Hawkins G Larkin L Kotila SM. An international outbreak of Salmonella enterica serotype Enteritidis linked to eggs from Poland: a microbiological and epidemiological study. Lancet Infect Dis. (2019) 19:778–86. 10.1016/S1473-3099(19)30047-7 31133519

[28] Waldram A Dolan G Ashton PM Jenkins C Dallman TJ. Epidemiological analysis of *Salmonella* clusters identified by whole genome sequencing, England and Wales 2014. Food Microbiol. (2018) 71:39–45. 10.1016/j.fm.2017.02.012 29366467

[29] EFSA Ea European Centre for Disease Prevention and Control and European Food Safety Authority: Multi-Country Outbreak of Salmonella Enteritidis Phage Type 8, MLVA Profile 2-9-7-3-2 and 2-9-6-3-2 Infections. Stockholm; Parma (2017).

[30] Inns T Ashton PM Herrera-Leon S Lighthill J Foulkes S Jombart T. Prospective use of whole genome sequencing (WGS) detected a multi-country outbreak of *Salmonella*\ Enteritidis. Epidemiol Infect. (2017) 145:289–98. 10.1017/S0950268816001941 27780484 PMC9507544

[31] Team RC A Language and Environment for Statistical Computing. Vienna: R Foundation for Statistical Computing (2017).

[32] Inns T Lane C Peters T Dallman T Chatt C McFarland N. A multi-country *Salmonella*\ Enteritidis phage type 14b outbreak associated with eggs from a German producer: 'near real-time' application of whole genome sequencing and food chain investigations, United Kingdom, May to September 2014. Euro Surveill. (2015) 20:21098. 10.2807/1560-7917.ES2015.20.16.21098 25953273

[33] Dallman T Inns T Jombart T Ashton P Loman N Chatt C. Phylogenetic structure of European *Salmonella*\ Enteritidis outbreak correlates with national and international egg distribution network. Microb Genom. (2016) 2:e000070. 10.1099/mgen.0.000070 28348865 PMC5320589

[34] Drummond AJ Suchard MA Xie D Rambaut A. Bayesian phylogenetics with BEAUti and the BEAST 1.7. Mol Biol Evol. (2012) 29:1969–73. 10.1093/molbev/mss075 22367748 PMC3408070

[35] Godbole GS Day MR Murthy S Chattaway MA Nair S. First report of CTX-M-15 *Salmonella*\ Typhi from England. Clin Infect Dis. (2018) 66:1976–77. 10.1093/cid/ciy032 29471386

[36] Doumith M Godbole G Ashton P Larkin L Dallman T Day M. Detection of the plasmid-mediated mcr-1 gene conferring colistin resistance in human and food isolates of Salmonella enterica and *Escherichia coli* in England and Wales. J Antimicrob Chemother. (2016) 71:2300–5. 10.1093/jac/dkw093 27090630

[37] Nair S Ashton P Doumith M Connell S Painset A Mwaigwisya S. WGS for surveillance of antimicrobial resistance: a pilot study to detect the prevalence and mechanism of resistance to azithromycin in a UK population of non-typhoidal Salmonella. J Antimicrob Chemother. (2016) 71:3400–8. 10.1093/jac/dkw318 27585964

[38] Kanagarajah S Waldram A Dolan G Jenkins C Ashton PM Carrion Martin AI. Whole genome sequencing reveals an outbreak of Salmonella Enteritidis associated with reptile feeder mice in the United Kingdom, 2012-2015. Food Microbiol. (2018) 71:32–8. 10.1016/j.fm.2017.04.005 29366466

[39] Nair S Patel V Hickey T Maguire C Greig DR Lee W. Real-time PCR assay for differentiation of typhoidal and nontyphoidal salmonella. J Clin Microbiol. (2019) 57:e00167–19. 10.1128/JCM.00167-19 31167843 PMC6663909

[40] Scaltriti E Sassera D Comandatore F Morganti M Mandalari C Gaiarsa S. Differential single nucleotide polymorphism-based analysis of an outbreak caused by Salmonella enterica serovar Manhattan reveals epidemiological details missed by standard pulsed-field gel electrophoresis. J Clin Microbiol. (2015) 53:1227–38. 10.1128/JCM.02930-14 25653407 PMC4365250

[41] Authority EFS Prevention ECFD Control Multi-Country Outbreak of Salmonella Enteritidis Infections Linked to Polish Eggs. EFSA Supporting Publications (2017). p. 1353E

[42] Yoshida CE Kruczkiewicz P Laing CR Lingohr EJ Gannon VP Nash JH. The *Salmonella In Silico* Typing Resource (SISTR): an open web-accessible tool for rapidly typing and subtyping draft *Salmonella* genome assemblies. PLoS ONE. (2016) 11:e0147101. 10.1371/journal.pone.0147101 26800248 PMC4723315

[43] Nguyen M Long SW McDermott PF Olsen RJ Olson R Stevens RL. Using machine learning to predict antimicrobial MICs and associated genomic features for nontyphoidal *Salmonella*. J Clin Microbiol. (2019) 57:e01260–18. 10.1128/JCM.01260-18 30333126 PMC6355527

[44] Nadon C Van Walle I Gerner-Smidt P Campos J Chinen I Concepcion-Acevedo J. PulseNet International: vision for the implementation of whole genome sequencing (WGS) for global food-borne disease surveillance. Eurosurveillance. (2017) 22:30544. 10.2807/1560-7917.ES.2017.22.23.30544 28662764 PMC5479977

[45] Alikhan NF Zhou Z Sergeant MJ Achtman M. A genomic overview of the population structure of Salmonella. PLoS Genet. (2018) 14:e1007261. 10.1371/journal.pgen.1007261 29621240 PMC5886390

[46] Pearce ME Alikhan NF Dallman TJ Zhou Z Grant K Maiden MCJ. Comparative analysis of core genome MLST and SNP typing within a European Salmonella serovar Enteritidis outbreak. Int J Food Microbiol. (2018) 274:1–11. 10.1016/j.ijfoodmicro.2018.02.023 29574242 PMC5899760

[47] Chattaway MA Greig DR Gentle A Hartman HB Dallman TJ Jenkins C. Whole-genome sequencing for national surveillance of *Shigella flexneri*. Front Microbiol. (2017) 8:1700. 10.3389/fmicb.2017.01700 28974944 PMC5610704

[48] Chattaway MA Schaefer U Tewolde R Dallman TJ Jenkins C. Identification of *Escherichia coli and Shigella*. species from whole-genome sequences. J Clin Microbiol. (2017) 55:616–23. 10.1128/JCM.01790-16 27974538 PMC5277532

[49] Chattaway MA Dallman TJ Gentle A Wright MJ Long SE Ashton PM. Whole genome sequencing for public health surveillance of Shiga toxin-producing *Escherichia coli* other than serogroup O157. Front Microbiol. (2016) 7:258. 10.3389/fmicb.2016.00258 26973632 PMC4776118

[50] Elson R Awofisayo-Okuyelu A Greener T Swift C Painset A Amar CFL. Utility of whole genome sequencing to describe the persistence and evolution of *Listeria monocytogenes* strains within crabmeat processing environments linked to two outbreaks of listeriosis. J Food Prot. (2019) 82:30–8. 10.4315/0362-028X.JFP-18-206 30702931

[51] Greig DR Schaefer U Octavia S Hunter E Chattaway MA Dallman TJ. Evaluation of whole-genome sequencing for identification and typing of *Vibrio cholerae*. J Clin Microbiol. (2018) 56:e00831–18. 10.1128/JCM.00831-18 30135231 PMC6204685

[52] Inns T Flanagan S Greig DR Jenkins C Seddon K Chin T. First use of whole-genome sequencing to investigate a cluster of *Yersinia enterocolitica*, Liverpool, United Kingdom, 2017. J Med Microbiol. (2018) 67:1747–52. 10.1099/jmm.0.000856 30325299

